# Universal Access to Advanced Imaging and Healthcare Protection: UHC and Diagnostic Imaging

**DOI:** 10.3390/medsci9040061

**Published:** 2021-09-27

**Authors:** Pietro Cappabianca, Gaetano Maria Russo, Umberto Atripaldi, Luigi Gallo, Maria Paola Rocco, Giovanni Pasceri, Michele A. A. Karaboue, Silvia Angioi, Salvatore Cappabianca, Alfonso Reginelli

**Affiliations:** 1 Centro Interdipartimentale di Ricerche Neurologiche, Università degli Studi della Campania “Luigi Vanvitelli”, 80121 Napoli, Italy; pietrocappabianca@gmail.com; 2 Dipartimento di Medicina di Precisione, Università degli Studi della Campania Luigi Vanvitelli, 80121 Napoli, Italy; gmariarusso@hotmail.it (G.M.R.); umbertoatripaldi@gmail.com (U.A.); luigigallo992@gmail.com (L.G.); mariapaolarocco@gmail.com (M.P.R.); salvatore.cappabianca@unicampania.it (S.C.); 3 Facoltà di Medicina e Chirurgia, Università Vita-Salute San Raffaele, 20132 Milano, Italy; giovannipasceri@pasceri.it; 4 Sezione Medicina Legale, Dipartimento Medicina Sperimentale “L. Vanvitelli”, Università degli Studi della Campania Luigi Vanvitelli, 80121 Napoli, Italy; mikelekaro@hotmail.it; 5 Dipartimento di Scienze Politiche “Jean Monnet”, Università degli Studi della Campania Luigi Vanvitelli, 80121 Napoli, Italy; silivaangioii@hotmail.it

**Keywords:** diagnostic imaging, installed CT fleet, radiology, UHC

## Abstract

Universal Health Coverage (UHC) is a set of principles adopted by the World Health Organization (WHO) aimed to guarantee access to primary care for the entire world population through a range of essential health services without neglecting the diagnostic aspect. Italy is one of the signatory states, which means that diagnostic services should be appropriated and exigible throughout the national territory equally. Our research analyzed and identified the main criticalities in terms of age, territorial distribution, and technological and health appropriateness of installed Computed Tomography (CT) needed to meet the principles of UHC. Data analyzed in our study were published by Assobiomedica at the end of 2016 and by COCIR, which included and investigated the installed fleet of diagnostic equipment in the Italian sanitary system and in various European countries. The 6th point of the Alma Ata Declaration defines the concept of “primary health care”, which includes the importance of the diagnostic phase in the Italian health care system to provide Essential Levels of Assistance (LEA). It is clear from our studies that the technology at the national level is not adequate to satisfy the UHC principles or the European criteria, with negative effects on the diagnostic standards and on advanced screenings. This study conducted on the installed CTs in Italy at the end of 2016 confirms the persistence of progressive aging that has been recorded for several years in the health facilities of the country and suggests incentive policies for the replacement of obsolete equipment, which represent a form of investment rather than a cost, due to the nature of the expenditure itself, one-off and amortizable over time.

## 1. Introduction

Universal Health Coverage (UHC) is a strategy internationally adopted by the World Health Organization (WHO), aimed to guarantee access to primary care for the entire world population [1,2,3,4,5].

Many countries are exploring opportunities and different ways to achieve UHC [6,7,8,9,10,11,12]. Italy ratified these agreements, which means that all people have access to health services, when and where they need them, without financial hardship. It includes the full range of essential health services, including the diagnostic aspect, from health promotion to prevention, treatment, rehabilitation, and palliative care.

Following the same political–ideological path, in 1978 the Alma Ata Declaration was signed and the State Parties committed to reach national health in coverage, emphasizing the need for effective and urgent action to develop and implement primary health care in every part of the world, in a spirit of technical cooperation. It is important to include the diagnostic aspect of healthcare in the sanitary system, in its dual value to monitor the evolution of a possible pathology and to assess the occurrence and risk factors in a timely manner to ensure the effectiveness of subsequent treatments.

Therefore, the Italian National Health System is essentially based on principles such as universality, equity, and solidarity, in order to guarantee all citizens the Essential Levels of Assistance (so called LEA) Universal Health Coverage (UHC).

Diagnostic Imaging plays an important role in the Essential Levels of Assistance for the diagnosis and screening of almost all the diseases spread throughout the area, especially those with the highest rates of incidence and lethality.

To ensure the principles of UHC regarding radiology, diagnostic services should be appropriated and exigible throughout the national territory equally.

Providing non-exigible LEA throughout the national territory breaks the principle of equality. It is also necessary to define these services’ suitability, which does not mean efficiency but a fair compromise among the effectiveness of the treatments, their safety, their cost, and the inclusion in a complex health system.

To guarantee that the entire population can benefit from adequate diagnostic services, it is necessary to have a homogeneous distribution throughout the territory, at the local and national levels, of diagnostic imaging equipment, which go hand in hand with the technological advancement we are currently witnessing.

An adequate distribution of diagnostic instruments throughout the territory not only allows all citizens to access health services, but it would also lead to a reduction in waste in public health by monitoring the maintenance costs of the most obsolete equipment, which have a strong impact on health expenditures.

Among the various diagnostic imaging instruments, our research focused on CT, given its considerable diffusion and usefulness in diagnosis, staging, and follow up. We analyzed and identified the main criticalities in terms of age, territorial distribution, and technological and health appropriateness of the installed CT needed to meet the principles of UHC.

## 2. Materials and Methods

Our survey analyzed the data of the research published by Assobiomedica at the end of 2016, which included an overall analysis of the installed fleet of diagnostic imaging in the Italian sanitary system (public and private structures), including CTs, divided into two macro-groups: CTs with fewer than 16 layers and those with a number equal to or greater than 16 layers (Table 1) [13].

The concept of “technological adequacy period”refers to the maximum age that an installed fleet can be considered adequate at the date of the survey compared to the technologies currently available on the market. For CT devices, this time is approximately 7 years [14,15].

We analyzed the study of the European Association representing companies in the COCIR sector (the European Coordination Committee of the Radiological, Electromedical and Healthcare IT Industry), which investigated the issue of the age of the installed fleet of diagnostic equipment in various European countries [16]. Through a careful reworking of the data obtained from this study, in order to verify the progressive aging of the technologyalready registered over the past years caused by a reduction in investment in medical technologies in Italy, we identified and analyzed the main critical issues in terms of age, technological adequacy, and obsolescence with respect to the state of the art.

For the comparison with Europe, we used the COCIR “golden rule”, common to the various technologies, as a guideline for the purposes of evaluation related to investment policies in Healthcare for these technologies, classifying as adequate an installed park that has:

- at least 60% of the equipment no older than five years, reflecting the current technological state, while still offering the possibility of being updated at reasonable cost;
- no more than 30% of the equipment between 6 and 10 years old, still suitable for use, but requiring the development of replacement strategies;
- no more than 10% of equipment older than 10 years, as it is no longer in line with the state of the art [16].

Additionally, we assessed the number and the geographical distribution of equipment present in Italian public and private structures compared to other European countries [16].

The geographical areas are divided into macro-areas and micro-areas.

According to the ISTAT definition:

The macro-areas included;

North;

Center;

South-Isles.

The micro-areas included:

For North: Piemonte, Valle D’Aosta, Lombardia, Trentino-Alto Adige/Sudtirol, Veneto, Friuli-Venezia Giulia, Liguria, Emilia Romagna;
For Center: Toscana, Umbria, Marche, Lazio;
For South and Islands: Abruzzo, Molise, Campania, Puglia, Basilicata, Calabria, Sicilia, Sardegna.

We evaluated the geographic distribution of CTs with respect to population density, dividing the population of a macro-area by the number of technologies present in the territory. This ratio allowed us to calculate the number of CTs per million inhabitants.

## 3. Results

The equipment found in all departments inthe years 2014, 2015, and 2016 is included in this statistical sample (Table 1).

The comparison of the presence of equipment in the various Italian macro-areas is calculated in Table 2.

In Table 3 we reported the geographical distribution of the installed equipment with respect to the population density; for almost all the technologies considered, there are generally no significant differences among the geographical macro-areas (Table 3).

Comparing the number of devices per million inhabitants from 2014 to 2016, there was only a slight increase in the distribution of tomographs to a number greater than or equal to 16 layers, from 23 to 25 per million inhabitants, over the time of this study (Table 4).

Comparing the average age of the technology installed and in use in Italy at the end of 2016to the same figure at the end of 2015, it is possible to see substantial aging for almost all the types of equipment under investigation (Table 5).

Furthermore, by analyzing each of the geographic macro-areas, a significant percentage of the installed technology does not meet the technological adequacy criteria defined above (Table 6 and Table 7).

In Table 8 we report the comparison between the number of devices at the end of 2016 compared to 2013 and 2009.

In Table 9 we report the percentage breakdown of the fleet installed in Italy at the end of 2016 according to three age ranges: up to 5 years (≤5); over 5 and up to 10 years (>5; ≤10); and over 10 years (>10). For comparison, the European average figure at the end of 2015 (COCIR) is shown in relation to the golden rule of COCIR.

## 4. Discussion

Universal access to advanced imaging helps to safeguard good health. The UHC is a global strategy internationally adopted in order to guarantee access to primary health care to the global population. This concept is noted in major international agreements, which have been ratified by Italy. In article 12 of the International Covenant on civil and political rights, signed in 1966, the State Parties committed to recognize the right of everyone to access the highest attainable standard of physical and mental health. The second paragraph of the article mentioned above specifically enlists the steps to be taken by the State Parties in order to fully achieve this right, among which some of the most relevant are the prevention, treatment, and control of epidemic, endemic, occupational, and other diseases (par 2nd c) and the creation of conditions which would ensure medical service to all and medical attention in the event of sickness [17].

The same political–ideologic path was followed by the Alma Ata Declaration in 1978, through which the State Parties committed to reach the goal of the universal health coverage, stressing the necessity of an urgent and effective plan to globally develop and improve primary health care with technical cooperation among the States.

The 6th point of the Alma Ata Declaration defines the concept of “primary health care”, stating that primary healthcare is essential health care based on practical, scientifically sound and socially acceptable methods and technology made universally accessible to individuals and families in the community through their full participation and at a cost that the community and country can afford to maintain at every stage of their development in the spirit of self-reliance and self-determination.

Given the definition mentioned above, it is clear that UHC must include the diagnostic phase of the health care process as an instrument both to control illness evolution and to evaluate the onset and the risk factors with proper timing to guarantee the efficacy of subsequent treatments. Access to the advanced imaging has raised numerous issues concerning the right to health care.

As stressed by several authors [18], the value of diagnostic information (so-called VODI) has always been given insufficient attention in scientific discourse raised to evaluate the public investments needed in order to fully achieve the right to health care. The main issue raised by the access to advanced imaging is that, unlike with therapeutic protocols, it is often harder to demonstrate its effectiveness, considering that the diagnostic protocols have direct influence on both the patient’s management and the efficiency of the National Healthcare System. However diagnostic protocols have shown their primary relevance in developing a system based on efficiency and accessibility.

As shown in our data, an analysis of the geographical distribution of the installed technology with respect to the population density, for almost all the technologies considered, shows that there are generally no significant differences among the geographical macro-areas. However, in case of CT devices with fewer than 16 layers, in the regions of central Italy the number of devices per million inhabitants is significantly higher than the relative national average. Comparing the number of devices per million inhabitants from 2014 to 2016, there was only a slight increase in the distribution of CT machines with greater than or equal to 16 layers, from 23 to 25 per million inhabitants, over the time of this study. An important parameter, in order to understand the criticalities regarding the distribution of this diagnostic equipment in the territory, is represented by the analysis of the average age of the installed technology in the various geographical macro-areas, which shows an almost homogeneous age picture of the territory, without particular deviations from the national average. From a comparison of the average age of the technology installed at the end of 2016 compared to the period of technological adequacy, it emerges that some technologies significantly exceed the age threshold, especially for conventional diagnostic equipment. In case of CTs with less than 16 layers, despite an average age of the installed technology almost at the limit of adequacy compared with the previous year, there is a decrease of those with ages falling within the adequacy threshold, so much so that 256 devices, more than half of those in use (54%), exceed the relative technological adequacy age limit (evaluated at seven years) and, therefore, it would be appropriate to consider their possible replacement. This is a snapshot that further confirms the need to consider the adoption of systemic approaches and policies aimed atreplacing obsolete equipment in a programmed manner. The breakdown of the installed technology by age group of equipment at the end of 2016 is compared with similar values observed at the end of the previous three years, both nationally and by analyzing each of the three geographical macro-areas. From the comparison it clearly emerges that in the case of CTs with 16 layers or more, the installed base has progressively aged over the years, a trend that can also be seen for CTs with fewer than 16 layers, although to a lesser extent. This indicates the natural aging of the equipment already installed as a result of the passage of time, but which obviously does not correspond to the installation of new equipment, for both replacement and the creation of new diagnostic points. It is important to analyze the lack of modernization of the installed technology, which is linked to the spending review and to the few investments of the regions aimed at a progressive renewal of the diagnostic equipment supplied. From the point of view of individual countries, the COCIR analysis shows a significant variability in terms of age profile: in the CT segment, for example, Italy andseveral other countries, including Spain, Ireland, Greece and Austria, have a significant negative trend compared to the standards of the golden rule. This is an element that raises considerable concern if we think of the dose reduction aspects that the new equipment makes possible in the execution of the diagnostic test. Conversely, countries such as France, Denmark, and Sweden have very high percentages (between 55% and 70%) of equipment aged less than five years (survey at the end of 2015). In France, this trend can realistically be traced back in large part to the reimbursement mechanisms of diagnostic services in the country, which are aimed at favoring the generational turnover of technologies, or to significant targeted investments.

The current and prospective situation of the technological park at national level is therefore not adequate according to the European Association’s criteria, with negative effects, for example in the case of CTs, on the possibility of adopting, through the upgrade of equipment, software for containment of the radiation dose for older equipment.

More specifically, the availability of technical machinery to perform advanced diagnostic screenings and to fulfill the protection standards required by Italian national law (so-called LEA) must be taken into account [19].

Such considerations are a logical consequence of the scientific and legal literature acknowledging the relevance of economic evaluations of the criteria used in order to guarantee the efficiency of health services. There are three key points to be considered in such an evaluation: measurement, quantification, and the cost-benefit ratio of diagnostic and therapeutic protocols of essential care provision. It is undeniable that the request for access to diagnostic tests is constantly increasing, given the accuracy and precision provided by scientific development in predicting onset and evolution of an illness. Nonetheless, there is currently a gap between public demand for diagnostic tests and the ones actually conducted, and there are several reasons for this. The first reason is the lack of specific items of expenditure in the national budget to be invested in diagnostic research activities [20]. The second reason concerns the unevenness of the diagnostic devices provided to the hospitals. Such unevenness is particularly evident if one considers the situation of Northern and Southern Italian hospitals. Healthcare facilities in the Northern Italy, by virtue of multiple socio-economic factors, are equipped with more advanced diagnostic devices compared to the Southern Italian ones. This consideration raises a controversial issue concerning informed consent [21,22]. Given that difference, one wonders if, when the patient provides his informed consent, he should be informed of the availability of more technologically advanced diagnostic devices in other healthcare facilities in the country. A preliminary distinction is considered necessary. If, due to the kind of illness and for the diagnostic perspectives offered by the available technology in other hospitals, such information may result in an increase of the chance of an accurate diagnosis, consequently increasing the probability of the patient’s healing, then such information should be disclosed. On the contrary, if, considering the illness, the use of a more advanced device is completely irrelevant, disclosing such information would be unnecessary. The implementation of such a practice, despite that distinction, however, might encounter applicative difficulties as it would require a general and prior knowledge of all the available devices available throughout the National territory. The SIRM (Italian Society of Medical Radiology) makes a complete list of such devices on a yearly basis. This consideration implies that, during the whole year, it would be complex, if not impossible, to be promptly updated about such information.

The correct enhancement of diagnostic imaging techniques in the hospital setting is based on a further consideration regarding the exposure to radiation in the medical field and the definition of the so-called Diagnostic Level of Reference (LDR). Directive 2013/59/Euratom requires Member States to adopt proper legislation concerning protection against the risks related to ionizing radiation. In such context, a primary role was played by ISS (Superior Institute of Healthcare), whose purpose was to provide adequate scientific support in order to properly impose such a directive.

That directive, in fact, flowed into Legislative Decree n. 101/2020. The European Union’s purpose was, with such a directive, to harmonize and standardize, in Member States, the interventional radiology procedures in terms of patients’ and healthcare workers’ radio-protection, considering the LDRs [23].

The regular review of both the LDRs and of the medical team appointed for the operation of diagnostic devices implies remarkable financial expenses charged to the National Healthcare System.

The aforementioned expenses, however, cannot always be afforded due to multiple factors, such as, in the Italian case, the enormous public debt accumulated in the previous years that limited of the amount of money invested in the National Healthcare System and, on an ideological level, induced reluctance to consider the relevance of diagnostic techniques from a functional point of view in order to protect the patients’ right to health. It is therefore necessary to achieve a greater number of CT scan per capita.

Taking as an example the experience of Japan, which is one of the nations with the highest number of diagnostic devices per capita, having a large number of devices can lead to an increase in the demand for inadequate diagnostic performance and therefore a greater exposure to radiation for the population.

This is a serious problem from the standpoints of avoiding unnecessary exposure and considering medical economics. In these circumstances, it is easy to see how important the leading role of radiology companies is in preparing diagnostic imaging guidelines. [24,25]. Concerning the exposure issue, it is easy to understand how having a state-of-the-art diagnostic park substantially reduces potentially harmful radiation while improving the overall image quality.

## 5. Conclusions

The study conducted on the installed CTs in Italy at the end of 2016 confirmed the persistence of progressive aging that has been recorded for several years in the health facilities of the country. The phenomenon concerns different types of equipment such as CT with 16 and higher layers, technology for which over the years there has been a progressive decrease in the percentage of equipment less than five years of age and the corresponding increase instead of those over 10 years old. Of particular concern is the number of devices still in operation that are older than 10 years. A critical issue is certainly the persistence of the trend of increasing technology age in the case of several of the technologies under investigation. The data indicate a failure to install state-of-the-art equipment, either for new diagnostic points or as replacements for the previous ones.

A systematic replacement process—even progressive over time—of the most obsolete diagnostic technologies, could lead to cost optimization and areturn on the initial investment in the short to medium term.

In this context, it is believed that incentive policies for the replacement of obsolete equipment represent a form of investment rather than a cost, due to the nature of the expenditure itself, one-off and amortizable over time.

## Figures and Tables

**Table 1:** Number of devices by class in 2016, 2015, and 2014 in Italian public and private structures (both subsidized and not).

Diagnostic Devices	2016	2015	2014
CT < 16 SLICES	474	471	470
CT ≥ 16 SLICES	1538	1470	1419
TOTAL	2012	1941	1889

**Table 2:** Breakdown of the installed equipment by aggregations in geographic macro-areas.

Diagnostic Devices	Italy	North	Center	South and Isles
CT < 16 SLICES	474	116	218	140
CT ≥ 16 SLICES	1538	650	313	575
TOTAL	2012	766	531	715

**Table 3:** Number of devices per million inhabitants (1 million inhabitants/ratio total CTs-total population) by aggregations in geographical macro-areas.

Diagnostic Devices	Italy	North	Center	South and Isles
CT < 16 SLICES	8	4	18	7
CT ≥ 16 SLICES	25	23	26	28
TOTAL	33	27	44	35

**Table 4:** Number of devices per million inhabitants (1 million inhabitants/ratio total CTs-total population). Comparison between the end of 2016, 2015, and 2014.

Diagnostic Devices	2016	2015	2014
CT < 16 SLICES	8	8	8
CT ≥ 16 SLICES	25	24	23
TOTAL	33	32	31

**Table 5:** Average age of the technology installed by macro geographical areas compared to date of first installation.

Diagnostic Devices	Italy	North	Center	South and Isles
CT < 16 SLICES	7, 5	8, 3	7	7, 6
CT ≥ 16 SLICES	6, 7	6, 6	7	6, 6

**Table 6:** Distribution of the technology installed at the end of 2016 in Southern Italy and the Islands. On the right the CT SCAN % in age intervals from the date of first installation. Comparison between the end of 2016 and the end of 2015 and 2014.

South and Isles	Diagnostic Devices			<5 Years			≥5; ≤10 Years			>10 Years		
	2016	2015	2014	2016	2015	2014	2016	2015	2014	2016	2015	2014
CT < 16 SLICES	140	133	130	18%	18%	21%	56%	56%	57%	26%	26%	26%
CT ≥ 16 SLICES	575	573	554	39%	39%	43%	47%	47%	43%	14%	14%	13%

**Table 7:** Distribution of the technology installed at the end of 2016 in Italy. On the right the CT SCAN % in age intervals from the date of first installation. Comparison between the end of 2016 and the end of 2015 and 2014.

Italy	Diagnostic Devices			<5 Years			≥5; ≤10 Years			>10 Years		
	2016	2015	2014	2016	2015	2014	2016	2015	2014	2016	2015	2014
CT < 16 SLICES	474	471	470	6%	25%	27%	67%	53%	57%	27%	22%	16%
CT ≥ 16 SLICES	1538	1470	1419	14%	35%	40%	66%	50%	48%	20%	14%	12%

**Table 8:** Comparison between the number of devices at the end of 2016 compared to 2013 and 2009. Data processed by the Ministry of Health include public and private structures.

Diagnostic Devices	2016	2013	2009
CT < 16 SLICES and ≥16 SLICES	2012	1950	1837

**Table 9:** Percentage breakdown of the fleet installed in Italy at the end of 2016 according to three age ranges: up to 5 years (≤5); over 5 and up to 10 years (>5; ≤10); and over 10 years (>10). For comparison, the European average figure at the end of 2015 (COCIR) is shown in relation to the golden rule of COCIR.

Diagnostic Devices	Assobiomedica Data Italy (2016)			COCIR Data Europe AVG (2015)			COCIR Golden Rule		
	≤5	<5; ≤10	≥10	≤5	<5; ≤10	≥10	≤5	<5; ≤10	≥10
CT < 16 SLICES and ≥ 16 SLICES	40%	39%	21%	48%	39%	13%	60%	30%	10%

## Data Availability

The data presented in this study are available in the article.

## Abbreviations

WHO	World Health Organization
CT	Computed Tomography
COCIR	European Trade Association representing the medical imaging, radiotherapy, health and electromedical industries
LEA	Essential Levels of Assistance
ISTAT	Istituto Nazionale di Statistica
VODI	Value of diagnostic information
LDR	Level of Reference
ISS	Superior Institute of Healthcare
